# The Reformed Public House

**Published:** 1923-12

**Authors:** A. W. Simons

**Affiliations:** 27, Meads Road, Wood Green, N. 22


### The Reformed Public House.

SIR,—Most of your readers who are not avowed Prohibitionists will agree with your correspondent, Mr. Dering, as to the advisability of reforming the public house, but many will object to the scheme he advocates—the extension of the Carlisle State ownership experiment to the whole of the country.

Apart from the tremendous financial liability which would necessarily have to be taken over by the State—and this, at the present time, is sufficient alone to put any such scheme out of court—it is doubtful whether the same partial success of the Carlisle experiment will be maintained when the whole of the licensed trade of the country is to be managed, instead of a few houses situated in one small spot. We have very many examples of what public houses should be that have been converted by their brewery owners. All that is necessary is that the Government should give the same facilities to private public house owners for the reform of their property as they have given to those responsible for the Carlisle experiment. If this were done, in the space of a few short years public house reform would become general, instead of a spasmodic movement. It is a well-known fact that many of the largest brewery companies are simply waiting for this official encouragement to be given, and once the larger breweries started wholesale remodelling of their property it would be only a very short time before the smaller breweries and licensees fell into line and remodelled their houses.

If the Government were to grant facilities for the passing of a measure on the lines of the Public House Improvement Bill it would have the desired effect, would not cause the outlay of a penny of the taxpayers' money, and, moreover, would be the means of providing employment for thousands in the general reconstruction of licensed houses that would ensue.

A. W. SIMONS.

27, Meads Road,

Wood Green, N. 22.

